# Supplementary material for: Emergent spatial goals in an integrative model of the insect central complex
Source: PLoS Comput Biol. 2023 Dec 18;19(12):e1011480. doi: 10.1371/journal.pcbi.1011480 (PMC10760860; doi:10.1371/journal.pcbi.1011480)
Supplement: S1 Text — Fig A. Virtual worlds used in simulations. Concentric circles on the ground do not appear during the simulations and are displayed here to show distance, 100l.u. separate consecutive circle radius. Vector memory replication of Le Moël et al [20] have been conducted in the empty environment. The sensory attraction to the innate green cylinder have been conducted in the single landmark. The MB route following simulation, both with a straight or zigzag route, have been conducted in both the enriched environment and the cluttered environment, without green landmarks in it. Finally, multiple source exploration simulations have been conducted in the cluttered environment, with randomly positioned green landmarks as food sources. Fig B. Eye model visual processing. We built an eye model with the aims to (A) reduce the resolution inherited from the raw simulation images and (B) represent the heterogeneity observed generally in insects eyes [99–101], i.e. the frontal and horizon part presenting often a higher resolution than the rest of the eye. (C) Each ommatidia is then assigned the set of pixel corresponding to its projection on the pyOpenGL rendering planes (4 orthogonally organized planes forming the panoramic view with a 160° vertical span. The activity rate of an ommatidia is calculated by the averaged light-level of all its assigned pixels. (D) The two color channels visible by insects (Green & Blue) are separated and can be used for different pathways. The Green channel is defined to create the frontal (above the horizon) visual field detection of green landmark (innate). Alternatively, the blue channel is used to input into the MB through the vPNs (learned). Fig C. CX model connectivity matrix. (A) Generic connectivity pattern used between individual neuron group. The ID of each neuron of a single type is based on the exitence of the functional columns observed in several CX sub-structure. (B) Overall connectivity matrix representing the whole CX model. Fig D. Comp [file pcbi.1011480.s001.pdf]

Supporting information

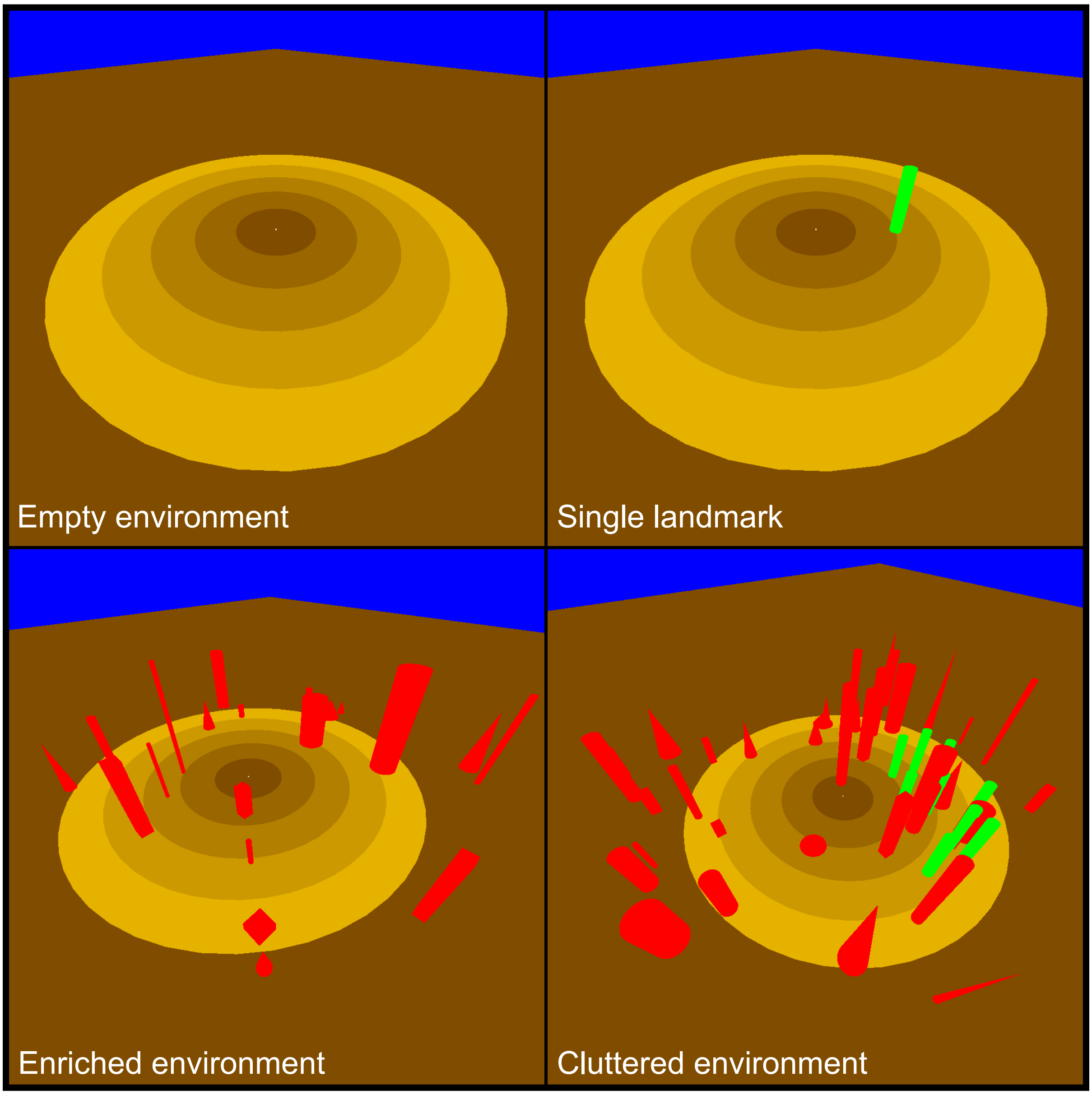

Fig A: Virtual worlds used in simulations.
Concentric circles on the ground do not appear during the simulations and are displayed here to show distance, 100l.u. separate consecutive circle radius. Vector memory replication of Le Moël et al [1] have been conducted in the empty environment. The sensory attraction to the innate green cylinder have been conducted in the single landmark. The MB route following simulation, both with a straight or zigzag route, have been conducted in both the enriched environment and the cluttered environment, without green landmarks in it. Finally, multiple source exploration simulations have been conducted in the cluttered environment, with randomly positioned green landmarks as food sources.

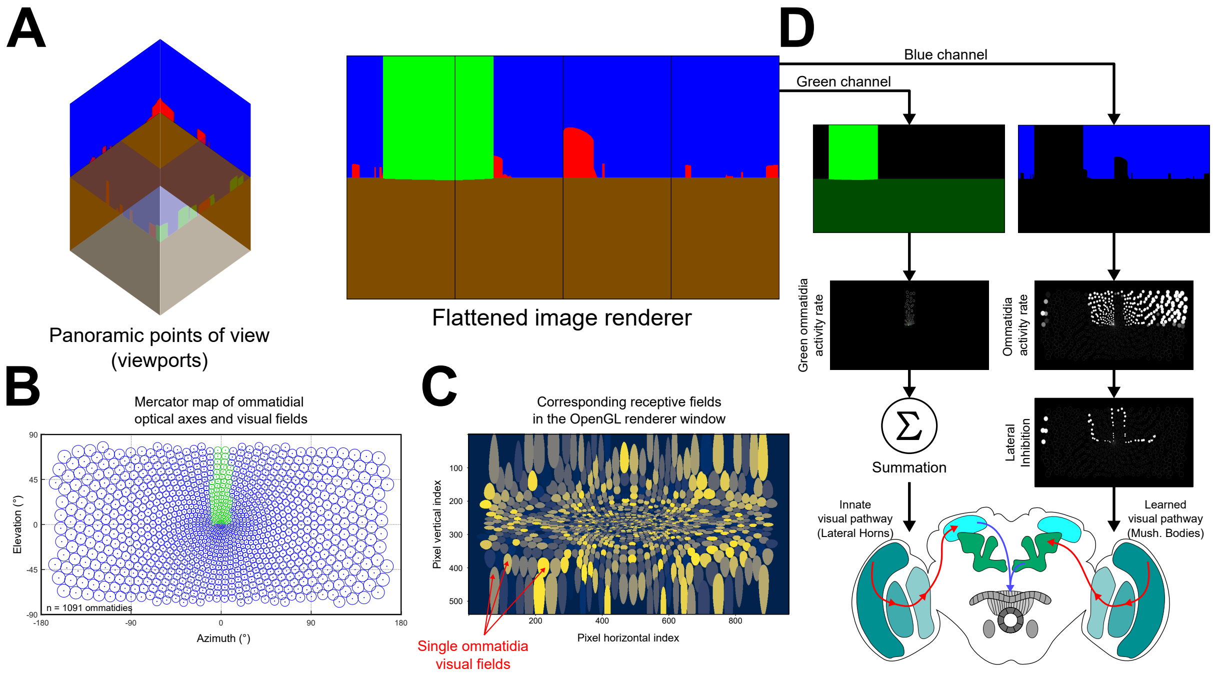

Fig B: Eye model visual processing.

We built an eye model with the aims to **(A)** reduce the resolution inherited from the raw simulation images and **(B)** represent the heterogeneity observed generally in insects eyes [2–4], i.e. the frontal and horizon part presenting often a higher resolution than the rest of the eye.

(C) Each ommatidia is then assigned the set of pixel corresponding to its projection on the pyOpenGL rendering planes (4 orthogonally organized planes forming the panoramic view with a 160° vertical span. The activity rate of an ommatidia is calculated by the averaged light-level of all its assigned pixels. **(D)** The two color channels visible by insects (Green & Blue) are separated and can be used for different pathways. The Green channel is defined to create the frontal (above the horizon) visual field detection of green landmark (innate). Alternatively, the blue channel is used to input into the MB through the vPNs (learned).

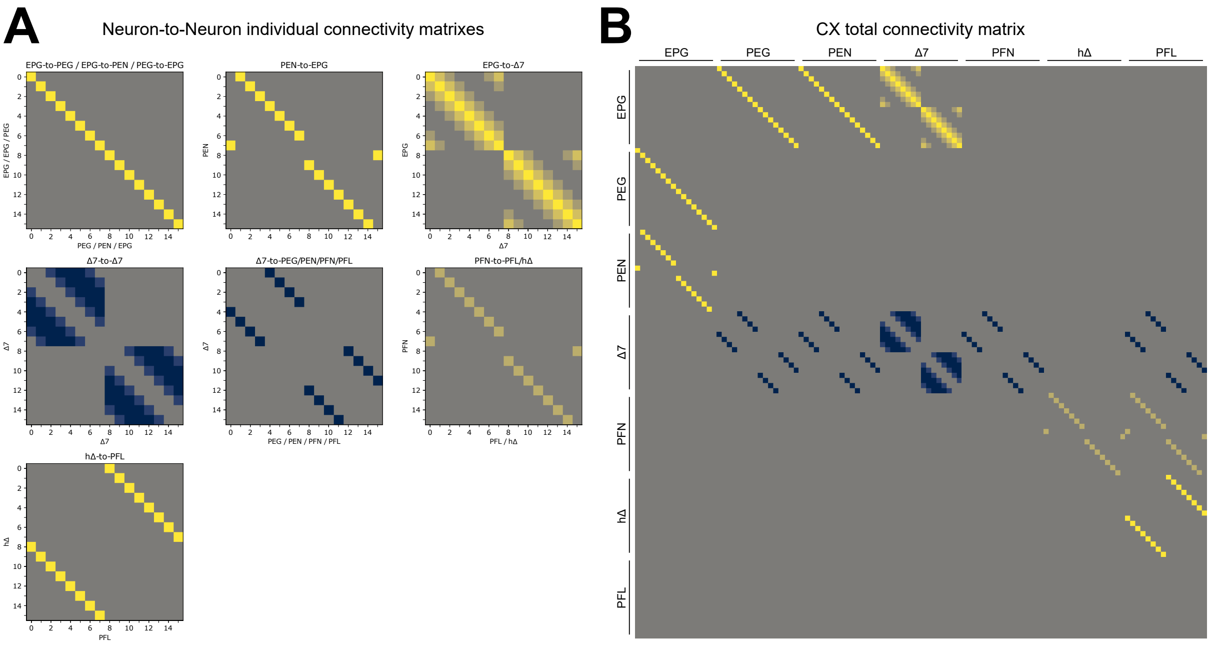

Fig C: CX model connectivity matrix.
(A) Generic connectivity pattern used between individual neuron group. The ID of each neuron of a single type is based on the exitence of the functional columns observed in several CX sub-structure.
(B) Overall connectivity matrix representing the whole CX model.

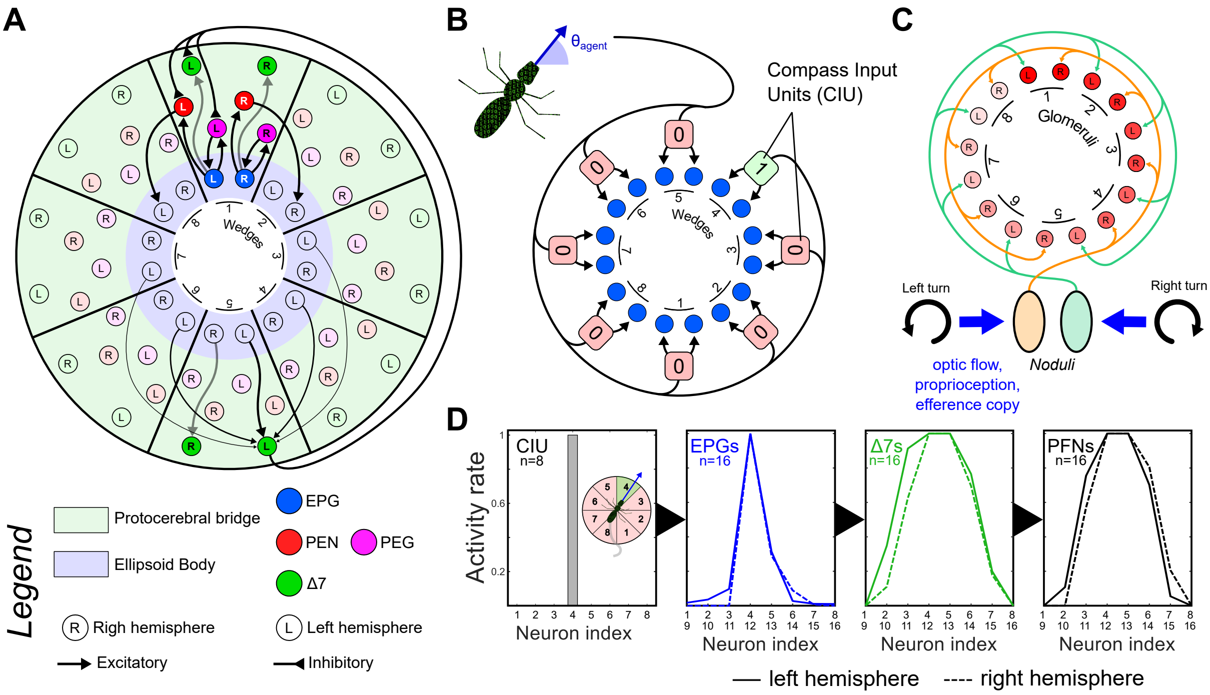

Fig D: Compass circuit.

The compass circuit consists of a ring attractor distributed between the EB and the PB that has been highlighted in recent neurophysiological studies in insects [5–7].

(A) Compass circuit diagram. The circuit is represented in a circular fashion to shed light on the columnar organization across EB and PB. The inter-neuron connectivity pattern is only shown for one functional column and repeated identically for every other. Note the intrinsic connectivity pattern across the PB of Δ7

(B) Compass orientation input to the *EPG* (EB). The orientation of the agent is compared with the preferred directions (with a 45° acceptance angle) of 8 orientation sensitive cells (Compass Neurons, CN), which could therefore correspond to mimic a sky polarization pathway. The cell that is sensitive to the current orientation is set with an activity rate of 1 while the others activity is set at 0. Each of this CN synapse to both *EPGs* of each wedge, one for each hemisphere, in the EB.

(C) Compass rotational input to the *PEN* (PB). Left/Right turns alternatively excite *PEN* on one hemisphere of the PB allowing the rotation of the compass according to the movement of the insect.

(D) Compass reformatting from the sensory input to the Δ7 layer and distribution to the *PFN* layer. Through the different layer of the compass circuit and particularly due to the Δ7 projection pattern, the single activity 'bump' inherited from the CN is transformed into a sinusoidal shape signal inherited by the *PFNs*.

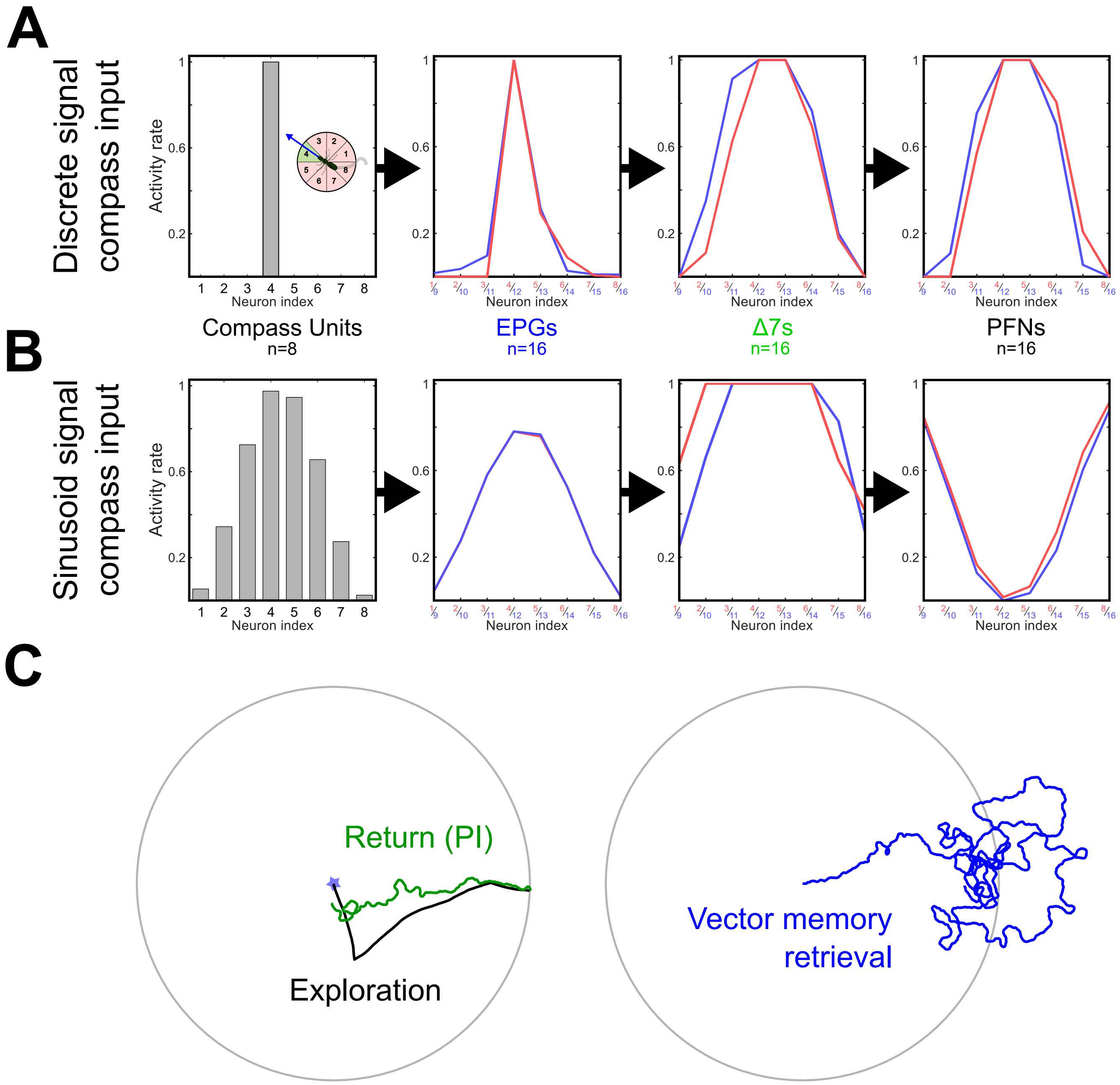

Fig E: Model with sinusoid compass inputs
(A) Head direction signal process from the Compass Units, following a winner take all activity rule, to the $PFNs$.
(B) Head direction signal process from the Compass Units, following a sinusoidal activity rule, to the $PFNs$. The activity of each units is calculated as the sinusoid of the difference between the orientation of the agent and of each compass unit preferred direction. $k_{\Delta 7}^{EPG}$ is adjusted to 0.4 to deal with the increase in overall activity across $EPGs$.
(C) Vector memory paradigm (Fig 4A) using the sinusoidal compass input.

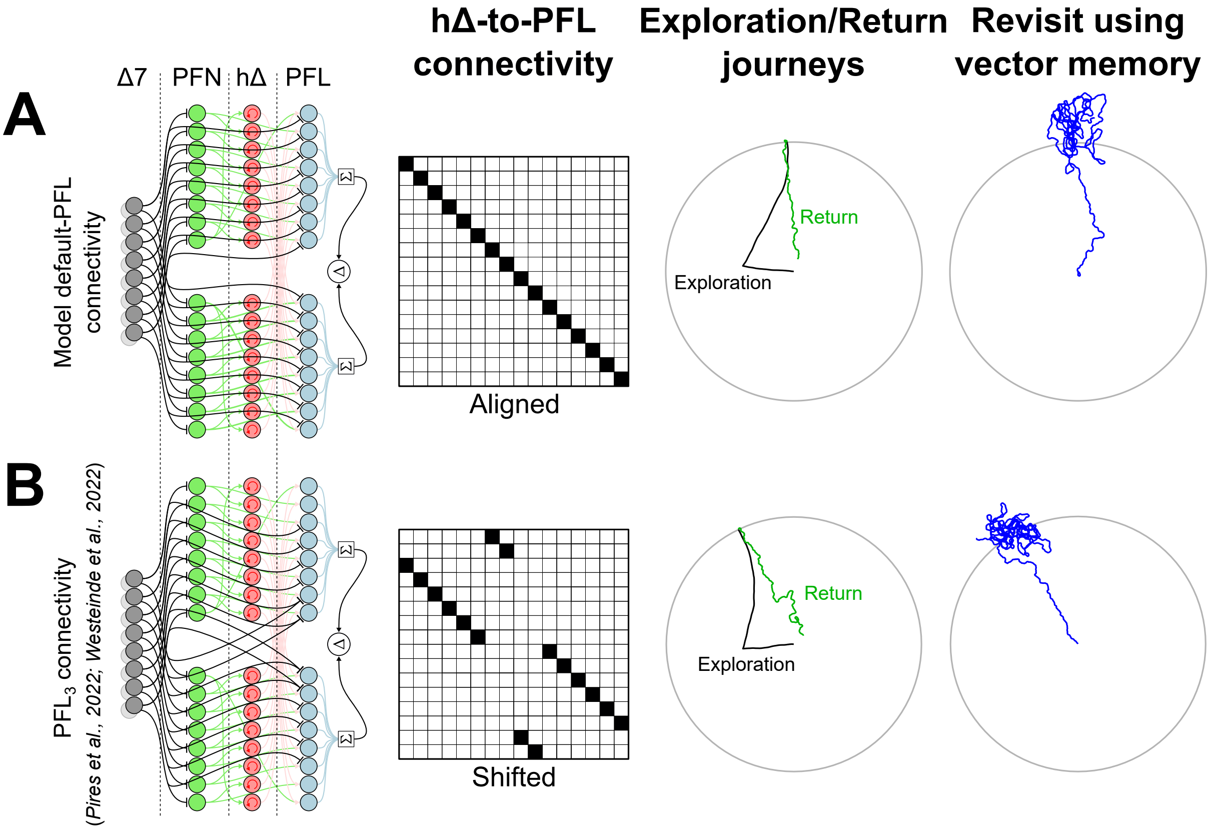

Fig F: Model with a realistic $\Delta 7-to-PFL_3$ connectivity pattern [8, 9].
(A) Default connectivity used in our model. No shift is applied from $\Delta 7$ to PFL as we used previously in [10].
(B) Connectivity pattern following a 2 columns shift from $\Delta 7$ to PFL_3 as observed in *Drosophila* [8,9,11]. Note that connectivity pattern are presented without the additional 4 columns shift inherited from the $\Delta 7$ projection pattern across the PB for simplicity.

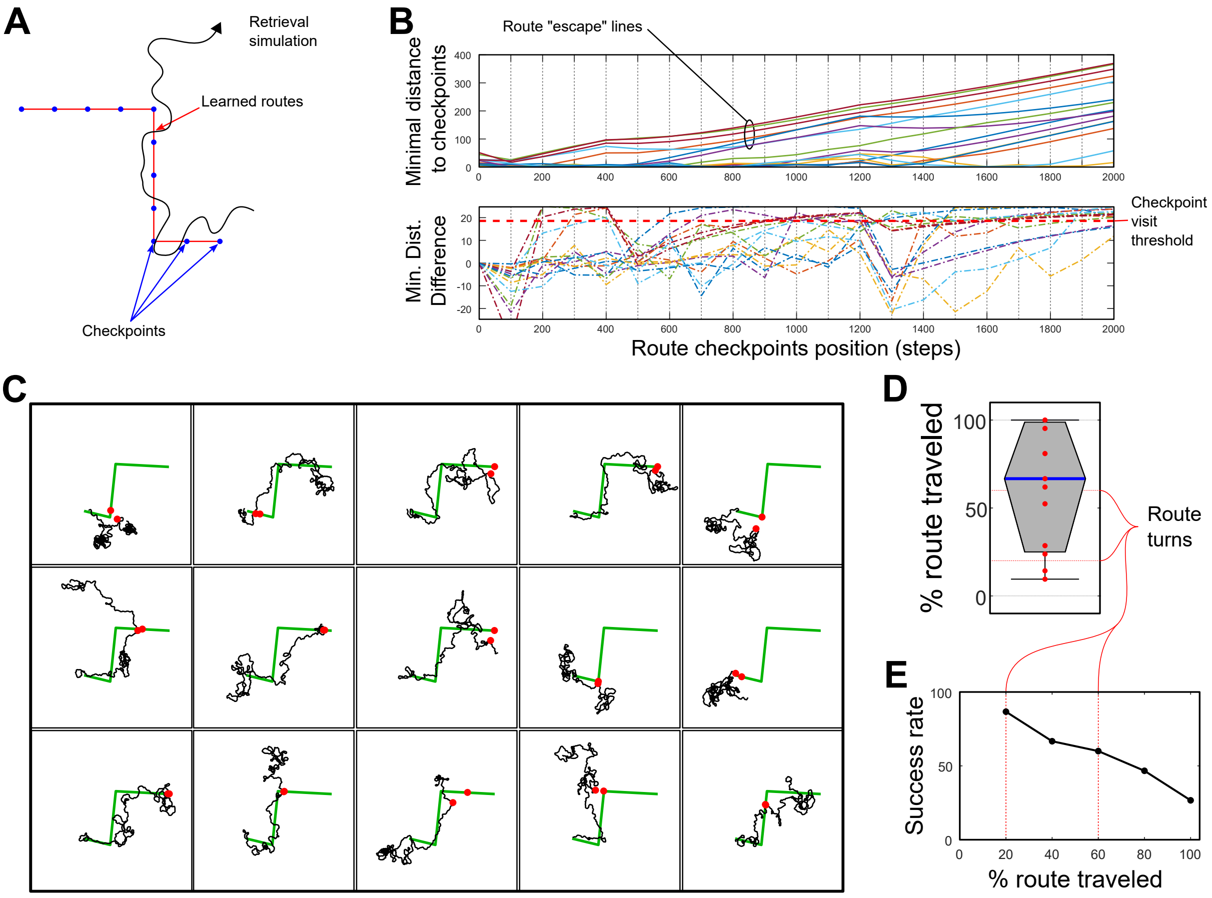

Fig G: Route following detection routine.

(A) Checkpoints are homogeneously distributed along the learned route.

(B) For each route following trial, we calculated a minimal distance to the checkpoints, each line indicate a route following attempt (n=15). Trials where the route is lost show typical escape line, which indicate a linear increase of the minimal distance to the checkpoints along the route whereas route following behaviour should be characterize by a constant (and lower) minimal distance to checkpoints. To identify this linear increase of the minimal distance to checkpoint we calculated its rate along checkpoints. We then defined a threshold based on the distance inter-checkpoints ($T_{rf} = 0.75D^{ck}$ with T_{rf} the threshold and D^{ck} the distance between two consecutive checkpoints). Whenever the minimal distance variation between two consecutive checkpoint failed to stay under this threshold ($Min(dCk^i) - Min(dCk^j) > T_{rf}$; with dCk the distance to a checkpoint $j = i + 1$), it indicates the agent never get closer than 0.75 times the distance inter-checkpoint than it was at the time it reach the minimal distance to the previous checkpoint, showing a lack of progress along the route. The checkpoint is then tagged as not visited and two consecutive checkpoints not visited are considered an end to the route following behaviour. The last checkpoint visited is therefore the further on-route location. Note that we did not consider any return to route following after it was consider out once.

(C) Example of the route following end point detection by the routine described previously on 15 trials of a simulation. Red dots indicate both the further checkpoint reached on the learned route (green line) and the closest location to this checkpoint on the retrieval attempt (black line).

(D) Boxplot of the percentage of route traveled based on the estimation of the last on-route checkpoint visited.

(E) Success rate (%) at different portion of the route. Note that the learned route turns always happen at 20% and 60% of the "zigzag" routes.

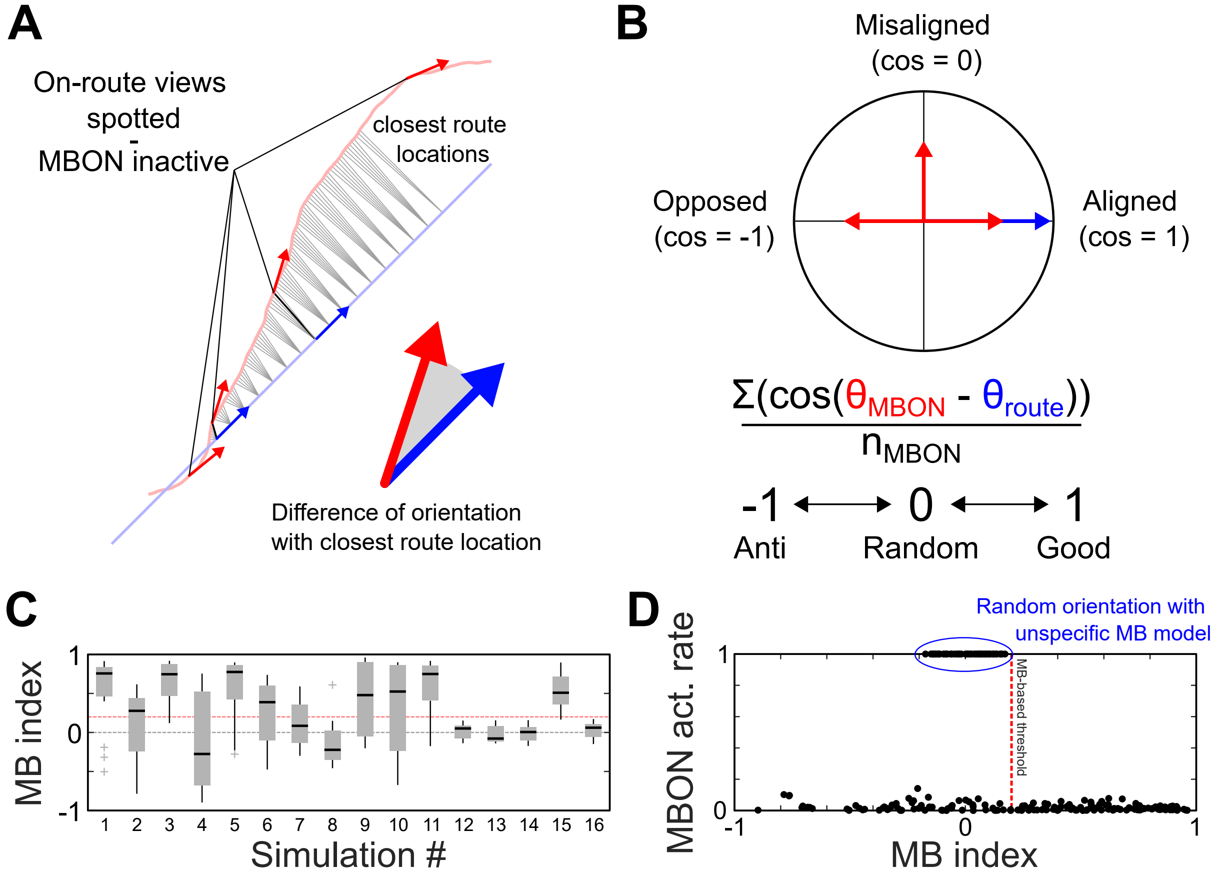

Fig H: Calculation of the MB model performance index.
(A) To estimate the intrinsic performance of the MB model in route following simulations, we define a performance index based on the alignment of the agent at time where the $MBON$ is active (views recognized as on the route) and the actual orientation of the nearest route location.
(B) Alignment of these orientation indicate a good recognition and the cosinus of the orientation difference lie close to 1, whereas perpendicular orientations lie around 0 and opposed alignement around -1. The averaging of all these comparison ($\frac{\sum cos(\theta_{MBON} - \theta_{route})}{n_{MBON}}$) therefore range from -1 for an overall anti-alignment between the actual route and the recognized route, to 1 for a perfect alignment/recognition.
(C) For each simulation we therefore estimate a score indicating the actual intrinsic performance of the MB model.
(D) To estimate a limit/threshold indicating that the MB model was better than random we benefit from simulations where the MB was actually constantly active, indicating an unspecific recognition of on-route views. We then arbitrarily define a value just over the range of MB performance indexes estimated for these simulations to define our threshold. All simulations with a score lower are considered impaired by the MB model itself and excluded from analysis.

	Outputs							
	$\mathbf{K_{out}^{in}}$	EPG	PEG	PEN	$\Delta 7$	PFN	$h\Delta$	PFL
**Inputs**	EPG	X	1.0	0.5	1.0	X	X	X
	PEG	0.6	X	X	X	X	X	X
	PEN	0.1	X	X	X	X	X	X
	$\Delta 7$	-0.2	-0.2	-0.2	X	-0.2	X	-0.6
	PFN	X	X	X	X	X	X	0.5
	$h\Delta$	X	X	X	X	X	X	0.5
	PFL	X	X	X	X	X	X	X

Table A: List of neuron-to-neuron gain parameters used in simulations (K_{output}^{input})
Note that parameters are not set to reflect a biological reality but rather to ensure a stable function of the model. However, no automatic optimization process have been applied to define this particular set of parameters.

References

1. Le Moël F, Stone T, Lihoreau M, Wystrach A, Webb B. The central complex as a potential substrate for vector based navigation. Frontiers in psychology. 2019;10:690.

2. Land MF, Eckert H. Maps of the acute zones of fly eyes. Journal of Comparative Physiology A. 1985;156:525–538.

3. Land MF. Visual acuity in insects. Annual review of entomology. 1997;42(1):147–177.

4. Straw AD, Warrant EJ, O'Carroll DC. Abright zone'in male hoverfly (Eristalis tenax) eyes and associated faster motion detection and increased contrast sensitivity. Journal of Experimental Biology. 2006;209(21):4339–4354.

5. Pfeiffer K, Homberg U. Organization and functional roles of the central complex in the insect brain. Annual Review of Entomology. 2014;59(1):165–184. doi:10.1146/annurev-ento-011613-162031.

6. Seelig JD, Jayaraman V. Neural dynamics for landmark orientation and angular path integration. Nature. 2015;521(7551):186–191. doi:10.1038/nature14446.

7. Kim SS, Rouault H, Druckmann S, Jayaraman V. Ring attractor dynamics in the *Drosophila* central brain. Science. 2017;356(6340):849–853. doi:10.1126/science.aal4835.

8. Pires PM, Abbott L, Maimon G. Converting an allocentric goal into an egocentric steering signal. bioRxiv. 2022;.

9. Westeinde EA, Kellogg E, Dawson PM, Lu J, Hamburg L, Midler B, et al. Transforming a head direction signal into a goal-oriented steering command. bioRxiv. 2022;.

10. Goulard R, Buehlmann C, Niven E Jeremy, Graham P, Webb B. A unified mechanism for innate and learned visual landmark guidance in the insect central complex. PLOS Computational Biology. 2021;17(9):e1009383. doi:10.1371/journal.pcbi.1009383.

11. Hulse BK, Haberkern H, Franconville R, Turner-Evans DB, Takemura S, Wolff T, et al. A connectome of the Drosophila central complex reveals network motifs suitable for flexible navigation and context-dependent action selection. bioRxiv. 2020;.
