# Supplementary material for: Emergent spatial goals in an integrative model of the insect central complex
Source: PLoS Comput Biol. 2023 Dec 18;19(12):e1011480. doi: 10.1371/journal.pcbi.1011480 (PMC10760860; doi:10.1371/journal.pcbi.1011480)
Supplement: Attachment — Submitted filename: PlosCB_responsereviewer.pdf [file pcbi.1011480.s002.pdf]

Reviewer #1: This is a very interesting computational/theoretical study that proposes a novel function for path integration in the insect central complex (Cx). While this well-documented behavioral capacity has previously been shown to allow central place foraging insects to return to their nests, the authors here propose that computation of a path integration vector could be combined with goal direction encoding to allow the insect to estimate the location of a goal in an allocentric framework. The basic idea (if I understand correctly) is that by combining internal representations of two vectors— one pointing towards a "home location" and one an estimate of the goal direction— an insect can estimate the location of a goal without the kind of place map observed in the vertebrate hippocampus. The authors demonstrate the plausibility of this scheme by building on their previously developed model of path integration in the Cx, and show that it improves navigational performance in two visually-guided tasks. Overall this is a fascinating and important new idea in insect navigation that makes several testable hypotheses. My comments largely have to do with clarifying presentation of some of the more difficult concepts (and checking if I have understood them correctly).

I had a couple of suggestions to enhance the clarity of the exposition:

1) Figure 1 dives already into complex anatomy of the insect brain. Perhaps it would be useful to introduce the main mathematical concept (in terms of vector representation and addition) as in Fig. 7 F near the top of the paper for readers who are less familiar with CX anatomy? We agree that the mathematical concepts of the model could be presented earlier in the paper to simplify the read. To this end, we added a figure (Fig. 2) that presents intuitively the model transformation from vectors into steering control and hopefully facilitates the reading.

2) Model exposition: this section was a bit tough to read and I wonder if it can be made a little easier on the reader. For example, each of sections 4.2.2, 4.2.3, and 4.2.4 begin by referencing previous work and then go straight to names of circuit elements followed by equations. I wonder if it would clearer for readers who are not familiar with this earlier work to start each section with a conceptual overview of what that part of the circuit does (e.g. for section 4.2.2., one could start with the material at line 175: "This circuit transforms the signal from a single activity bump…") then describe the components of the circuit and how they interact in words, then show the equations. The most challenging part of the manuscript for me was 4.2.4, but I thought a nice conceptual overview was given in the legend to Fig. 3C. Could this be brought to the top of the section and then unpacked to show how it is implemented in the model? Also in this section, could the authors spell out a bit more clearly what role the ßPFN and ßhΔ parameters play? It would be helpful here again to have a conceptual overview before the parameters are introduced. We added introductory sentence(s) to each subsection of the model to explain the purpose of the different model subparts and ease their understanding:

"The compass circuit is designed to transform external information, acquired by diverse sensory pathways described in insects, into an inner sinusoidal function representing the immediate orientation of the agent at any time." (l.182-184)

"The steering circuit is primarily designed to compute the error between the compass and goal vectors [31,52] and generates an asymmetrical signal that is decoded as the turning force for the agent (Fig. 2). In addition, it holds the two navigation vectors, the heading and homing vectors, that are used to generate the behaviour through the vector memory (see next section)." (l.196-199)

"The vector memory circuit is designed to store memory of either hΔ, carrying the PI homing vector, or PFN activity rate, carrying the immediate head direction vector. A reward signal induces the copying of one or both of these vectors into memory by modulating the synaptic weight of FBt inputs to the corresponding neuron type (hΔ or PFN). The memory can then be used later, under the control of an associated contextual signal that gates FBt activity." (l.228-232)

The role of the two parameters is now presented prior to the mathematical equations: "Because the length of the vector encoded is dependent on the population sinusoid signal (Fig. 2B), we use two gain

parameters, respectively β_{PFN} and $\beta_{h\Delta}$, to modulate the amplitude of the memory encoded at the level of the FB_t synapses. These parameters can be set over a range of values to test their effect on the simulated behaviour." (l.252-255)

a few typos or grammar fixes:

line 240: the term that modulate Done

line 446: Despite, while both model show Done

A few questions for the Discussion:

— Do I understand correctly that in this study the vector memories are stored in FBt synapses but have their effect by acting on connections from hΔ or PFN to PFL? It seems that some FBt neuron target axonal regions of hΔ cells and some target dendritic regions. What might be the function of those targeting dendritic regions? FBt dendritic projections would have no effect in our model because neurons are modeled as linear function and the subtraction would be done the same way (eq. 4). However, a more realistic approach suggests that dendritic connections would influence downstream computation in the neurons, such as the accumulation that subserves PI in our model. We can speculate that this could be used to reset the PI (to zero or to specific value, cf. Discussion), based on the FBt activity, or to gate/modulate the ongoing process of PI (when the orientation is uncertain/unknown for example). By contrast, axonal projections are ideal to subserve the influence of vector memory or similar by influencing the cell output without interference in the cell computation. We have added a brief discussion of this point to the text:

"Note here that in addition to axonal connections, used in our model to stably maintain the vector memory, FB_ts present projection to the dendritic region of hΔ subtypes [60] that are not considered in our model, due to the single linear unit neuron model. It remains to show what function(s) they could serve but we can speculate that dendritic projection would interfere with the inner computation of the neuron, and particularly the PI in the case of hΔ, as we hypothesise here. Therefore, introducing them in the model could be useful to set/reset the PI at a specific state (based on sensory/memory information, zeroing at the feeder in drosophila for example [25]) or eventually gating it (when the orientation/odometry become uncertain for example)." (l.623-630)

— line 717-719 notes that in this circuit a sensory memory cannot be recalled based on the PI state. However, the connectome does contain "FS" neurons that project from the FB back up to regions near the output of the MB. These have not been much studied but could be a basis for additional interactions between the spatial positioning system and the sensory memory system. Any pathway from the CX to sensory and/or memory neuropils would represent an interesting target to study the interplay between PI and sensory/memory that a cognitive map would presuppose. However, it necessitates a proper exploration that we cannot provide in this paper. We added a sentence to mention the existence of this pathway in the final paragraph.

Reviewer #2: This paper seems to me a very worthwhile exploration of how exhaustive modelling of the currently known circuitry in the central complex can account for a wide array of different navigational properties. It emphasises in particular the enormous importance of path integration in insect navigation and is well suited for publication in PlosS Computational Biology. I am a biologist rather than a computational modeler. On the assumption that many readers will also be biologists, I can best help by pointing out where the paper is a little difficult to follow. The earlier PLOS Computational Biology in 2021 paper by some of the same authors (ref 26) was more reader friendly.

ABSTRACT

Line 8: 'This transforms' is a bit unclear, 'Path integration transforms' might be better. Done, replaced by "The allocentric reference frame given by path integration" which is more precise.

Line 10: 'across insect species' could be broader and include crustaceans, perhaps arthropod – a term

that is used later. While the central complex is indeed conserved across arthropods, differences are substantial outside the insects and nothing is known about the circuits of this brain region outside insects. We replace the later mentioned term arthropods (l.60) to restrain the scope of the paper to insects.

AUTHOR SUMMARY

Line 12: 2 or 3 dimensional spatial problems 3 dimensional doesn't come up elsewhere so seems odd to have it in the summary (only examples that I know come from spiders, jumping spiders do 3D PI D.E.Hill and Portia M.Tarsitano) Done withdrawn 3-D mentions in the paper

Line 13: In this paper, we modelled a neural pathway that sustains insect visual-guided navigation both to a 'might neural circuitry be clearer? Done, replaced by "we modelled a neural network, based on the central complex connectivity".

INTRODUCTION

Great first para.

Lines 10-11:Perhaps reword: 'high structural conservation across species [10, 11]' to and its structure is highly conserved across species ? Done

Lines 42 -48: Passage below is quite hard to follow in an introduction. Refer to Fig 1 in the Intro?

'In addition to this positioning system, the projection geometry of some CX neurons effectively permits mental rotation of directional inputs. The virtual 180° shift carried by hΔb cells subserves the transformation of the allocentric orientation, from the head direction circuit, into an egocentric representation of the insect's holonomic motion [16, 17].'

We rewrote the paragraph in a less technical manner focusing on the vectorial manipulation and its importance in the CX:

"In addition to this positioning system, the CX hosts circuits that effectively generate, transform and use navigation vectors: the projection geometry of intrinsic neurons appears well suited to compute vector rotation and vector addition. Consequently, vectorial operations allow the computation of some of the circuit properties, for example the virtual 180° shift observed in the FB subserves the transformation of the allocentric head direction into an egocentric representation of the insect's traveling direction [16, 17]. The same principle applied to wind compass input allows Drosophila to reverse their up-wind/down-wind behaviour based on the odour context [18]." (l.43-48)

Fig.1 is now referenced in the introduction (l.38).

Line 52 'efficient trap-lines between multiple feeders' Explain trap-lines (rewarded locations visited in a set order)? We added the following specification for the trap-lines: "i.e. the visit of multiple feeders in a predictable and often optimised sequence" (l.54).

Line 57 Say a little more about central place foragers – social insects like ants and bees that live in a nest and from which foragers etc. We added the following sentence following the mention of central place forager: "species like wasps, bees or ants inhabiting a nest, for which efficient relocation of the nest and food sources by individuals is crucial to survival" (l.60-61).

Lines 66-73 do not read well. This section is also the first time FB is mentioned with no description or fig We rewrote the paragraph to make it more accessible and mention the role proposed recently for the fan-shaped body:

"The ability of the CX to generate and maintain navigation vectors to sustain an oriented behaviour even in absence of new sensory information is particularly adapted to support menotactic behaviours. It is, in addition, supported by the wide range of sensory streams converging to the CX, particularly in the fan-shaped body (FB) [15, 29–31]. This CX substructure has been proposed to be the centre of the comparison between the insect's own orientation (compass) and its current goal orientation (Fig. 2). The coexistence of multiple directional vectors, potentially representing competing goals, in the CX raises the question of their interactions together and with PI [32–34] to generate consistent and optimized behaviour [35]." (l.71-79)

MODEL

Lines 88-90 . It might help to give a brief account of 'ring attractor' and 'bump of activity'. We added the following sentence to define the two terms:

" i.e. a circuit that generates a stable representation of a circular variable, here the immediate heading direction. This head direction circuit generates an activity 'bump', in the form of a sinusoidal activity pattern across the population of neurons (EPGs) that tracks the animal's allocentric heading [9, 37]." (l.95-97)

Figure 1 is excellent but has no abbreviations so in the legend give full wording with abbreviations in parenthesis, e.g. Ellipsoid body (EB) Figure 1 caption now includes the full wording of all abbreviations cited there.

Line 94 Explain how inhib neurones (ER), as shown in Fig 1, carry directional info We added the following description of the ER function:

"The orientation of the different sensory compasses are dynamically imprinted, linking the egocentric orientation of a specific cue (visual, wind, ...) with a stable inhibitory pattern, in a unified reference frame [48]. ER neurons modulate downstream EP G population activity to generate an integrative inner compass." (l.103-106)

In addition, we added the following reference that present a detailed and functional model of the ring neurons function:

[48] *Mitchell R, Shaverdian S, Dacke M, Webb B. A model of cue integration as vector summation in the insect brain. Proceedings of the Royal Society B. 2023;290(2001):20230767.*

100-103 It makes sense but would be easier to follow if it were unpacked a bit. We simplified the description as follow:

"This particular geometry allows the comparison, at the PFL neurons level, of the current heading, carried by the direct inhibitory connection from Δ7 to PFL, with outputs from the PFN and hΔ, both excitatory, carrying the desired heading, e.g. goal direction (Fig. 2). These two pathways can be used alternatively in the model to define this goal vector based either on the head direction circuit [26] or on the PI [13]." (l.113-115)

Fig 5A Probably my lack of insight but I don't get panel C. We have modified the caption to make panel C clearer.

Fig S2 legend should E be D? Done

Line 382. Is ref 71 good evidence for degradation of route learning in CX. The claim in the abstract is 'Thus, CX lesions had a specific impact on learnt visual guidance' In the experiments the route is implemented by facing in the right visual direction and then being guided by a PI memory. So isn't break-down expected because of interference with PI. Shouldn't one therefor examine whether the lesions disrupt where the ant faces at the very start of its path? Our claim that the PI is directly involved in immediate sensory navigation indeed predicts that learnt visual guidance should be impacted by CX

lesions. However, in this paper, the experiments have been conducted indoors without providing celestial compass cues (such as polarized light or an artificial sun), therefore the ants’ behaviour is unlikely relying on PI. It is not clear to us how the lesion of the CX could be used to directly support or not our claim as both pathways, PI and sensory navigation, would be probably equally impacted by the lesions. Regardless, the reference here to this previous work only aims to justify the existence of a visual memory pathway from the MB to the CX for which we believe it is well-suited.

Incidentally, in the same vein, I like the modelling of how PI can aid route following.
